# The behaviour of ^226^Ra in high-volume environmental water samples on TK100 resin

**DOI:** 10.1007/s10967-017-5203-4

**Published:** 2017-03-13

**Authors:** E. M. van Es, B. C. Russell, P. Ivanov, M. García Miranda, D. Read, C. Dirks, S. Happel

**Affiliations:** 1 National Physical Laboratory, Hampton Road, Teddington, Middlesex TW11 UK; 2 Chemistry Department, University of Surrey, Guildford, Surrey GU2 7XH UK; 3 Triskem International, 3 rue des champs Géons, ZAC de l’Eperon, 35170 Bruz, France

**Keywords:** ^226^Ra, Extraction chromatography, TK100 resin, High volume water samples

## Abstract

Accurate, low-level measurement of ^226^Ra in high volume water samples requires rapid pre-concentration and robust separation techniques prior to measurement in order to comply with discharge limits and drinking water regulations. This study characterises the behaviour of ^226^Ra and interfering elements on recently developed TK100 (Triskem International) extraction chromatography resin. Distribution coefficients over a range of acid concentrations are given, along with an optimised procedure that shows rapid pre-concentration and separation of ^226^Ra on TK100 resin is achievable for high volume (1 L) water samples without the need for sample pre-treatment.

## Introduction

^226^Ra (half-life 1600 (7) years [1]) is a naturally occurring radionuclide in the ^238^U decay series, with additional inputs into the environment as a result of mining, the extraction of fossil fuels and chemical processing. It is the most significant contributor to occupational radiological doses arising from industrial naturally occurring radioactive material (NORM) sources [2–5]. ^226^Ra decays by alpha emission, with a maximum decay energy of 4.79 MeV (94.0%) [1], and gamma emission with a maximum decay energy of 186.2 (13) keV (32.8%). ^226^Ra is measurable by alpha spectrometry [6–8], liquid scintillation counting [9–11], gamma spectrometry [12–14] and inductively coupled plasma mass spectrometry (ICP-MS) [15–23]. Measurement of ^226^Ra is important with regards to drinking water quality as well as environmental monitoring around nuclear and industrial sites, including mining and processing sites for coal, phosphogypsum and uranium. The measurement of ^226^Ra is also important for radiological protection in areas of high radon concentration and more recently, in the measurement of produced and discharged waters following hydraulic fracturing (fracking) of shale gas resources [24, 25]. Consequently, there is a growing need to develop methods that can achieve rapid and accurate measurement of ^226^Ra in water samples from a variety of sources.

The World Health Organisation proposes a guideline value of 1 Bq L^−1^ for ^226^Ra in drinking water [26], whilst Canada specifies a Maximum Acceptable Concentration of 0.5 Bq L^−1^ [27]. The European Union gives an indicative annual dose of 0.1 mSv [4], and goes on to state that the method used for analysis must be capable of determining an activity concentration of 0.04 Bq L^−1^. In the United States, the Maximum Contaminant Level is set at 5 pCi L^−1^ (0.19 Bq L^−1^) for ^226^Ra and ^228^Ra combined [28]. Different limits apply to industrial discharges. The Environmental Permitting Regulations for England and Wales [3] stipulate maximum activity concentrations for discharging aqueous liquids to environmental water courses; in the case of ^226^Ra, the limit is 0.01 Bq L^−1^.

For all applications, separation and pre-concentration of ^226^Ra is required prior to measurement. A number of chemical separation techniques have been successfully applied to ^226^Ra separation, including anion and cation exchange, diffusive gradients in thin films (DGT), adsorption onto manganese dioxide resin, and extraction chromatography using strontium resin [19, 29–32]. A recently developed extraction chromatography resin is TK100 (Triskem International), which was developed primarily for extraction of ^90^Sr from high volume water samples [33]. The advantage of the resin is that ^90^Sr shows good retention when water is directly loaded at pH 2–8, compared to Sr-resin (Triskem International) where moderate to high acid concentrations are required prior to loading [34]. The TK100 resin consists of the same Sr-resin crown ether group that shows high selectivity for Sr (Di-t-butyl dicyclohexyl-18-crown-6), with the addition of Di(2-tethyl-hexyl) phosphoric acid (HDEHP). Whilst the resin has been characterised for ^90^Sr and several other radionuclides and stable elements [33–35], there is no information on the behaviour of ^226^Ra on TK100 resin. The potential to directly load high volume water samples with minimal prior treatment will offer a significant reduction in procedural time compared to alternative separation techniques. The resulting higher sample throughput would be highly advantageous in several applications including, routine monitoring of drinking water samples and compliance testing of discharges against regulatory targets.

The aim of this work is to further characterise TK100 resin, focusing on ^226^Ra in high volume (1 L) water samples. The behaviour of ^226^Ra and multiple other elements are investigated, as is the effect of high salt loadings on the resin performance. The ultimate aim is to produce a separation scheme that is applicable to both radiometric and mass spectrometric measurements of environmental or industrial water samples.

## Experimental

### Reagents and materials

Standard element solutions (Ba, Ca, Ce, Cs, Eu, La, Mg, Nb, Sr, Th, U, Y, and Zr) at starting concentrations from 1000-10,000 mg L^−1^ were purchased from Fluka Analytical and Fisher Scientific. NaCl, KCl, FeCl_2_, MgCl_2_, CaCl_2_, SrCl_2_ and BaCl_2_ for simulated fracking waters were all provided by Fisher Scientific. Nitric and hydrochloric acids were purchased from Fisher Scientific (Trace Analysis Grade). Standards and acids were diluted with ultrapure water (18 MΩ, <5 μg L^−1^ Total Organic Carbon) from an ELGA purelabflex water purification system. ^226^Ra standards were prepared at NPL over an activity concentration range of 0.01–10 Bq g^−1^. TK100 resin (100–150 μm particle size) was provided by Triskem International.

### Instrumentation

All measurements were performed using an Agilent 8800 triple quadrupole inductively coupled plasma mass spectrometer (ICP-QQQ-MS). The instrument is equipped with a collision-reaction cell positioned between two quadrupole mass filters, and was run in Single Quad mode (i.e. only one quadrupole mass filter operating) throughout. The instrument was fitted with a quartz double-pass spray chamber, MicroMist nebuliser, quartz torch (2.5 mm internal diameter), and nickel sample and skimmer cones. The instrument was tuned daily using a mixed 1 μg L^−1^ standard solution, and then tuned further for ^226^Ra. The sensitivity was on the order of 330 counts per second for a 1 ng L^−1^ (36.6 mBq g^−1^) solution, with a detection limit of ~0.1 ng L^−1^ (0.4 mBq g^−1^). A more detailed account of the performance of ICP-QQQ-MS for ^226^Ra measurement is described elsewhere [36].

### Calculation of distribution coefficients

The distribution coefficients for ^226^Ra and other group 2 elements for solutions at a range of acid concentrations (0.01–10 M HCl or HNO_3_) were calculated using Eq. 1:

1 $$K_{\text{d}} = \left( {\frac{{\left[ {{\text{CPS}}_{\text{i}} - {\text{CPS}}_{\text{f}} } \right]}}{{{\text{CPS}}_{\text{f}} }}} \right)\left( {\frac{V}{m}} \right)$$

where CPS_i_ and CPS_f_ are the initial and final counts per second measured by ICP-QQQ-MS for the element of interest, *V* is the volume of solution (mL), and *m* is the mass of the resin (g).

A 2 mL mixed stable element solution was prepared at a concentration of 100 μg L^−1^ for each element. The solution was evaporated to dryness, before being made up in 2 mL of HCl or HNO_3_ over a concentration range of 0.01–10 M. Prior to the addition of TK100 resin, a 0.5 mL aliquot was taken for ICP-MS measurement of the initial counts per second (CPS_i_). One hundred milligrams of TK100 resin was then added to each sample and left overnight. The sample was then filtered through a Whatman 0.45 μm filter paper, a 0.5 mL aliquot was taken for ICP-MS measurement of the final counts per second (CPS_f_). During analysis, ^232^Th was run as an internal standard in the instruments dedicated internal standard line to correct for instrument drift.

To assess the impact of high salt loading on resin performance, a range of single element solutions associated with fracking waters were prepared based on information from Maxwell et al. (Table 1) [25]. The solutions were prepared in 0.01 M HNO_3_ and the *K*_d_ values were calculated and compared to the results for non-salt loaded solutions.

**Table 1:** Concentration of elements used in simulated fracking water samples

Element	Source	Concentration range (g L^−1^)
Na	NaCl	40–400
K	KCl	1–100
Fe	FeCl_3_	1–100
Mg	MgCl_2_	1–100
Ca	CaCl_2_	10–1000
Sr	SrCl_2_	5–500
Ba	BaCl_2_	10–1000

### Elution profiles for high volume water samples

Optimal load and elution conditions determined from the *K*_d_ results were applied to blank water samples spiked with the range of elements listed previously. A 2 mL TK100 column (4 cm length × 0.5 cm diameter) was conditioned with 10 mL of the same acid concentration as the load solution, with no additional clean-up steps. A mixed stable element solution (1 L total volume at a concentration of 100 μg L^−1^) was loaded onto the column, followed by an elution volume of 10 mL. The load solution was split into 20 × 50 mL fractions, and the elution fraction split into 10 × 1 mL fractions to generate sample load and elution profiles, respectively. For all the fractions, a 1 mL aliquot was taken and diluted to 10 mL with 2% (v/v) HNO_3_ for ICP-QQQ-MS measurement to calculate the distribution coefficient and recovery of each element. The separation was carried out using a vacuum box at a flow rate of approximately 2 mL/min, with the procedure for a batch of 12 samples completed in approximately 8 h.

## Results and discussion

### Distribution coefficients

**Fig. 1:**
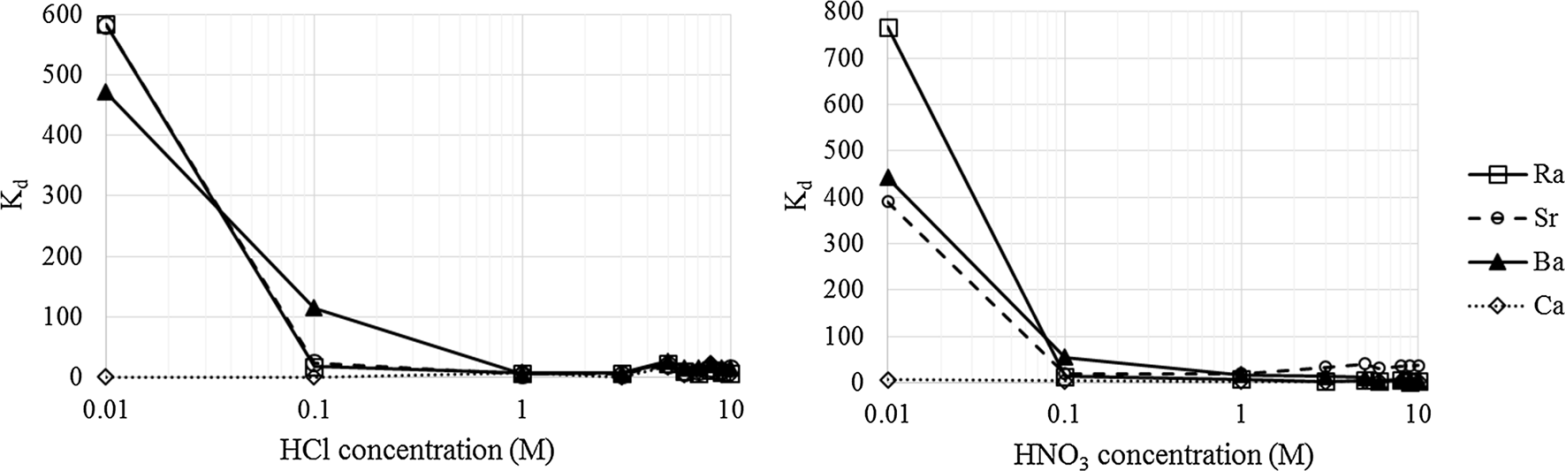
*K*_d_ values for Ra and other group 2 elements on TK100 resin in HCl and HNO_3_

Radium is well retained on the resin at pH 2, with *K*_d_ values of 5.8 × 10^2^ and 7.7 × 10^2^ for HCl and HNO_3_, respectively (Fig. 1). This compares to values of 5.8 × 10^2^ and 3.9 × 10^2^ for Sr. The distribution coefficient reduces significantly from 0.01 to 0.1 M for both HCl and HNO_3_; therefore, it is advised that the solution is kept at pH 2 prior to loading to avoid Ra losses. Nevertheless, the results confirm that direct loading of water samples acidified to pH 2 is applicable with respect to ^226^Ra analysis. Elution is achievable from 1 to 10 M HNO_3_ or HCl, with a maximum *K*_d_ of 2.4 × 10^1^ over this concentration range. The retention of Ra on TK100 resin is a noticeable improvement compared to Sr-resin, where Ra is not retained under any conditions; this may offer advantages where both strontium and radium isotope measurements are required from the same sample.

Calcium showed no retention on the resin under any of the conditions tested, which is beneficial for alpha spectrometry measurements where high Ca content may impact on the source preparation and reduce the resolution of the alpha spectrum, increasing the measurement uncertainty and producing unresolvable peaks. Barium and strontium performed similarly to Ra, in good agreement with the manufacturer’s claim that both elements would be retained at pH 2 and eluted in 8 M HNO_3_ and 2–3 M HCl for Ba and Sr, respectively [35]. As the acid concentration increases from 0.01 to 0.1 M, the *K*_d_ for Ra reduces by a factor of 56, which is similar for both HCl and HNO_3_. This compares to factors of 19 for Sr and 8 for Ba. Therefore, loading at pH 1 rather than pH 2 will adversely impact the retention of all these elements, with the effect most significant for Ra. The results suggest that that separation of Ra from Sr and Ba using TK100 resin would be challenging. For high volume water samples, loading conditions of 0.01 M HNO_3_/HCl and Ra elution in 1 M HNO_3_ and HCl for high volume water samples are investigated below.

### Elution profiles for high volume water samples

**Fig. 2:**
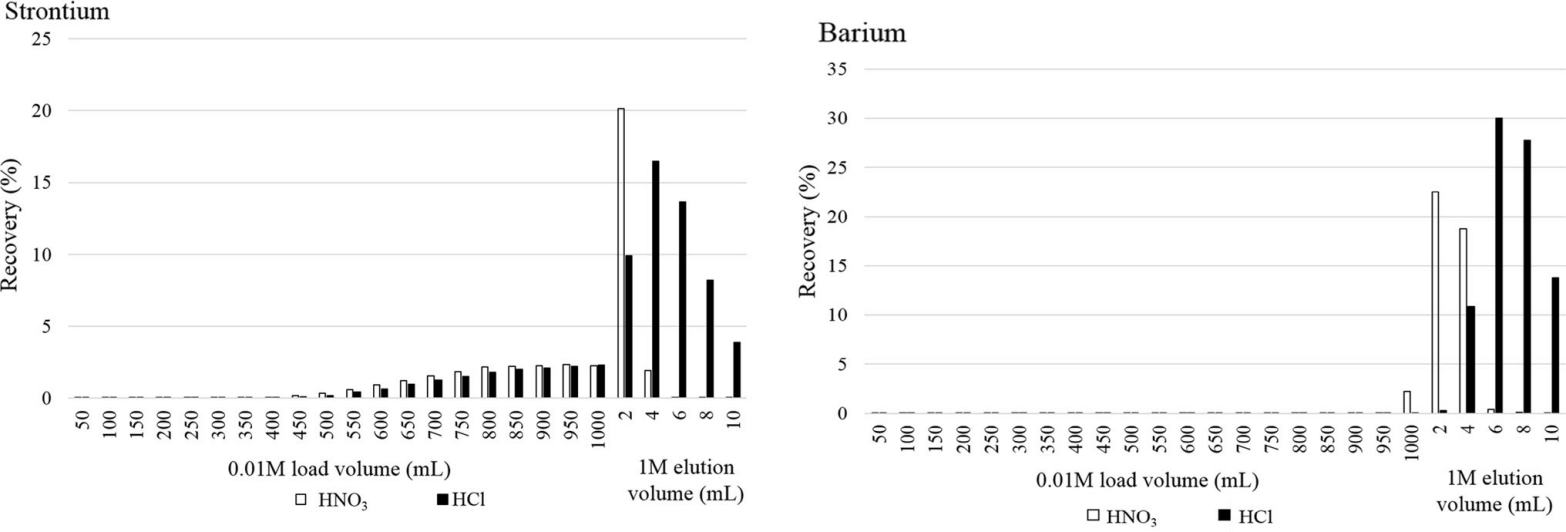
Elution profiles for Sr and Ba on TK100 resin in HNO_3_ and HCl conditions

The results for a multi-element solution showed good agreement between this study and results from Triskem regarding the behaviour of Sr, with breakthrough of Sr above a load volume of approximately 500 mL [35] (Fig. 2). Under both 0.01 M HCl and HNO_3_ loading conditions, breakthrough was <1% after a volume of 500 mL, but increased steadily at higher volumes, with loss of 18 and 15% after loading in 1 L 0.01 M HNO_3_ and HCl, respectively. Magnesium and calcium were not retained in the load solution.

Barium was well retained in the load solution, and both Ba and Sr were eluted in 1 M HCl or 1 M HNO_3_ along with ^226^Ra. This is an important consideration for ICP-MS applications, where polyatomic ^138^Ba^88^Sr can overlap with ^226^Ra [23]. Results from Triskem suggest that Sr is retained in 8–10 M HNO_3_, and that Ba is retained in 1 M HNO_3_ if the elution volumes are carefully controlled. By comparison, ^226^Ra is eluted in 1–10 M for both acids tested. To improve separation, a Sr-resin (Triskem International) column could be connected in tandem to a TK100 column following the sample load. If the elution conditions are changed to 3 M HNO_3_, then ^226^Ra, Ba and Sr will be eluted from the TK100 column; however, only Ra will be eluted from the Sr-resin column. The results in Fig. 2 also suggest that Sr, Ba and Ra recovery could be improved by increasing the elution volume from 10 to 20 mL.

Of the elements investigated, U, Th, Y, Zr, Nb, La, Ce and Eu, were also retained in the load solution, whilst Cs was not. Of these, U, Th, Y, Zr, and Nb were also well retained in 1 M HNO_3_ or 1 M HCl, therefore separation of ^226^Ra from naturally occurring radionuclides and other elements of importance to the nuclear industry is achievable using TK100. By comparison, La, Ce and Eu were detected in both eluents tested (not shown in Fig. 2), with higher recoveries in 1 M HCl (77–81%) compared to 1 M HNO_3_ (23–37%). This must be considered for ICP-MS procedures, where polyatomic ^87^Sr^139^La and ^86^Sr^140^Ce overlap with ^226^Ra. The formation of these interferences can be minimised by eluting in HNO_3_ rather than HCl, due to the lower La, Ce and Eu recovery, whilst Sr can be removed through the use of Sr-resin as previously described. Results from Triskem show that Pb is well retained when loaded at pH 2, but can be eluted along with U in 6 M HCl [35]. ^226^Ra is also eluted under these conditions, therefore separation of ^226^Ra from Pb and U is achievable by washing the column with 1 M HNO_3_ or HCl, followed by 6 M HCl to elute U.

### Impact of fracking waters on TK100 performance

**Fig. 3:**
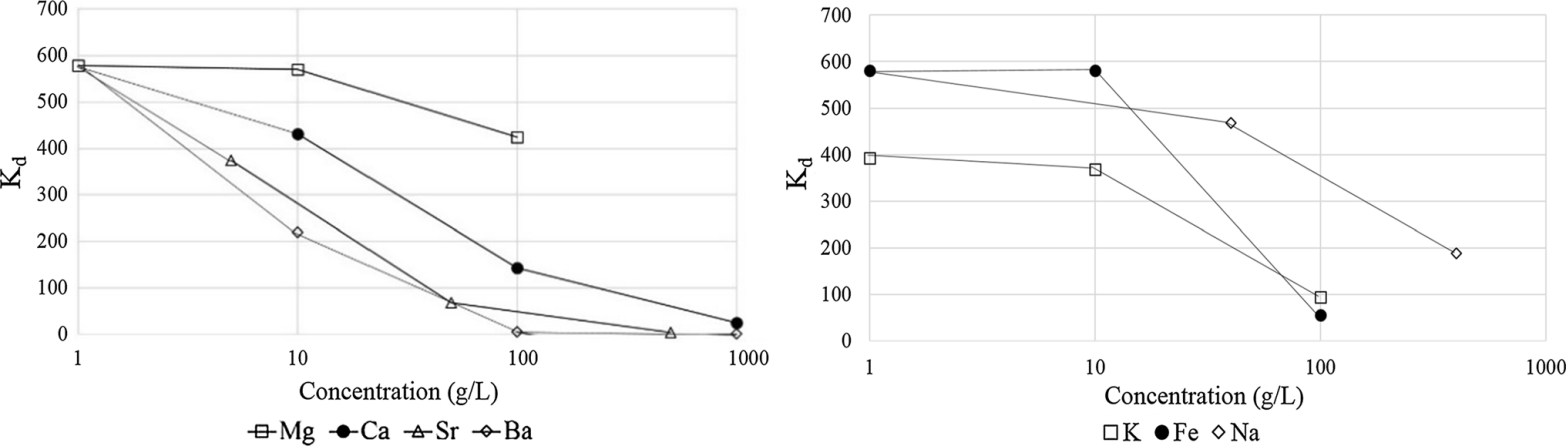
Impact of increasing salt loading on performance of TK100 resin for ^226^Ra retention at pH 2

As well as elevated activities of ^226^Ra, waters from fracking sources can contain high concentrations (g L^−1^) of multiple elements in the form of salts (including chlorides of Na, Ca, Mg, Sr and Ba) [25], which can impact the performance of chemical separation techniques. The performance of TK100 resin may be affected by the high matrix content associated with fracking waters, necessitating additional separation prior to sample loading. TK100 resin has been tested for Sr at pH 7 in the presence of 11,500 mg L^−1^ Na, 400 mg L^−1^ K, 1300 mg L^−1^ Mg and 500 mg L^−1^ Ca, with good retention of Sr under all conditions [33, 35]. Figure 3 shows the impact of a range of concentrations of Na, K, Fe, Mg, Ca, Sr and Ba chlorides (Table 1) on the retention of ^226^Ra at pH 2 (0.01 M HNO_3_). All of the salts tested adversely affect Ra retention on the resin, with SrCl_2_ and BaCl_2_ having the most significant impact; the *K*_d_ for Ra reduced at concentrations of 5 g L^−1^ SrCl_2_ and 10 g L^−1^ BaCl_2_. At the highest concentration of each element tested, the *K*_d_ for Ra ranged from <1 (1000 g L^−1^ BaCl_2_) to 425 (100 g L^−1^ MgCl_2_). Therefore, the sample matrix significantly impacts the performance of TK100 resin with regards to ^226^Ra, in agreement with the findings of Nelson et al. for other separation procedures [24].

## Conclusion

Rapid and accurate assessment of ^226^Ra is important with regards to drinking water quality, environmental monitoring and in measurement of produced waters from industrial processes including fracking. Radiometric and mass spectrometric determination of ^226^Ra requires pre-concentration and separation from interfering stable and radioactive isotopes prior to measurement. The application of recently developed TK100 extraction chromatography resin for ^226^Ra has been investigated for high volume water samples. The distribution coefficients were determined for ^226^Ra and interfering elements in HNO_3_ and HCl media, with the optimal conditions tested for 1 L water samples. Loading conditions of 0.01 M HNO_3_ or HCl were found to be suitable for retention of ^226^Ra, suggesting that samples can be loaded onto the resin with minimal sample pre-treatment, whilst elution is achievable in 1–10 M HNO_3_ or HCl. The high salt concentration of multiple elements associated with fracking waters adversely affects the performance of TK100 resin with Sr and Ba having the most significant impact; therefore, chemical separation prior to measurement must be considered for this application. Overall, TK100 resin provides a novel and rapid approach that is applicable as a single-stage or as part of a multi-stage pre-concentration and separation procedure for ^226^Ra in high volume water samples that can be used in conjunction with radiometric or mass spectrometric measurement.
